# Detection of SARS-CoV-2-RNA in post-mortem samples of human eyes

**DOI:** 10.1007/s00417-021-05529-x

**Published:** 2021-12-28

**Authors:** Josef Penkava, Maximilian Muenchhoff, Irina Badell, Andreas Osterman, Claire Delbridge, Florian Niederbuchner, Sarah Soliman, Martina Rudelius, Alexander Graf, Stefan Krebs, Helmut Blum, Michael Ulbig, Carmen Baumann, Daniel Zapp, Mathias Maier, Oliver T. Keppler, Chris P. Lohmann, Stephan Ledderose

**Affiliations:** 1 Department of Ophthalmology, Technical University Munich, Munich, Germany; 2 Max von Pettenkofer Institute & Gene Center, Virology, National Reference Center for Retroviruses, LMU München, Munich, Germany; 3 German Center for Infection Research, Partner Site Munich, Munich, Germany; 4 Department of Pathology and Neuropathology, TUM School of Medicine, Technical University Munich, Munich, Germany; 5 Department of Internal Medicine, Kliniken Südostbayern, Trostberg, Germany; 6 Department of Pathology, Ludwig-Maximilian University Munich, Munich, Germany; 7 Laboratory for Functional Genome Analysis, Gene Center, Ludwig-Maximilians-University, Munich, Germany

**Keywords:** COVID-19, SARS-CoV-2, Conjunctivitis, Vitreous sample, Intraocular involvement

## Abstract

**Purpose:**

To detect SARS-CoV-2 RNA in post-mortem human eyes. Ocular symptoms are common in patients with COVID-19. In some cases, they can occur before the onset of respiratory and other symptoms. Accordingly, SARS-CoV-2 RNA has been detected in conjunctival samples and tear film of patients suffering from COVID-19. However, the detection and clinical relevance of intravitreal SARS-CoV-2 RNA still remain unclear due to so far contradictory reports in the literature.

**Methods:**

In our study 20 patients with confirmed diagnosis of COVID-19 were evaluated post-mortem to assess the conjunctival and intraocular presence of SARS-CoV-2 RNA using sterile pulmonary and conjunctival swabs as well as intravitreal biopsies (IVB) via needle puncture. SARS-CoV-2 PCR and whole genome sequencing from the samples of the deceased patients were performed. Medical history and comorbidities of all subjects were recorded and analyzed for correlations with viral data.

**Results:**

SARS-CoV-2 RNA was detected in 10 conjunctival (50%) and 6 vitreal (30%) samples. SARS-CoV-2 whole genome sequencing showed the distribution of cases largely reflecting the frequency of circulating lineages in the Munich area at the time of examination with no preponderance of specific variants. Especially there was no association between the presence of SARS-CoV-2 RNA in IVBs and infection with the variant of concern (VOC) alpha. Viral load in bronchial samples correlated positively with load in conjunctiva but not the vitreous.

**Conclusion:**

SARS-CoV-2 RNA can be detected post mortem in conjunctival tissues and IVBs. This is relevant to the planning of ophthalmologic surgical procedures in COVID-19 patients, such as pars plana vitrectomy or corneal transplantation. Furthermore, not only during surgery but also in an outpatient setting it is important to emphasize the need for personal protection in order to avoid infection and spreading of SARS-CoV-2. Prospective studies are needed, especially to determine the clinical relevance of conjunctival and intravitreal SARS-CoV-2 detection concerning intraocular affection in active COVID-19 state and in post-COVID syndrome.


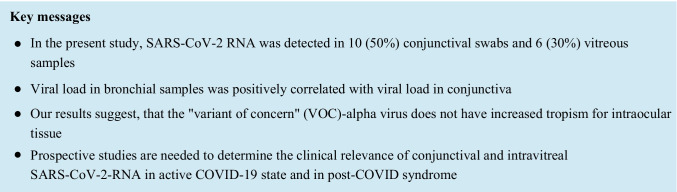


## Introduction

Since late December 2019, COVID-19 caused by the SARS-CoV-2 virus has become a global pandemic affecting humanity in an unprecedented way [1]. Since then, knowledge about the disease has rapidly expanded thanks to intensive research. It became apparent that COVID-19 is not only a pulmonary disease but can affect almost all other organ systems [2].

Ocular involvement has been reported in several studies. Eye symptoms occur in approximately one third of patients with SARS-CoV-2 infection, particularly in those with more severe disease [3, 4]. The most frequent ocular manifestation of SARS-CoV-2 infection is conjunctivitis, which may occur as a possible initial manifestation of COVID-19 [5–9]. In addition, retinal changes such as cotton-wool spots and retinal microhemorrhages have also been described [5, 10, 11]. Whether these symptoms result from direct infection of the eye, immunologic reactions, or ischemic damage to the visual system is not clear [5].

SARS-CoV-2 is mainly transmitted via aerosols and binds to the angiotensin-converting enzyme-2 (ACE-2) receptor to invade the host cell [12]. Involvement of the ocular surface may reflect high expression of the ACE-2 receptor in the conjunctiva and cornea [13, 14]. The ACE-2 receptor has also been detected in the aqueous humor and retina [15, 16]. Thus, direct infection of the eye by SARS-CoV-2 is theoretically conceivable.

Data on intraocular SARS-CoV-2, however, are still sparse with contradictory results in the available small post-mortem studies. For example, in one case report, SARS-CoV-2 RNA was detected in conjunctival swabs, whereas anterior chamber and vitreous samples were negative [17]. Another study showed a low prevalence of SARS-CoV-2 RNA in conjunctival and vitreous samples from COVID-19-positive decedents [18]. Casagrande et al. detected viral RNA of SARS-CoV-2 in the human retina of COVID-19 patients post mortem [19]. In contrast, no conjunctival, corneal, or intraocular SARS-CoV-2 RNA was found in other post-mortem studies [20–23].

SARS-CoV-2 involvement of the ocular surface is highly relevant particularly when planning and performing ophthalmic surgery, as infectious aerosols can be generated during ophthalmic procedures in SARS-CoV-2 positive patients [24]. The detection of viral RNA in the intraocular milieu may also provide a clue to the pathogenesis of ophthalmologic symptoms during COVID-19-related intraocular disease [25]. We therefore investigated in our post-mortem study whether SARS-CoV-2 RNA can be detected in the conjunctiva and vitreous body.

## Materials and methods

### Post-mortem sample collection

Between December 2020 and May 2021, we performed post-mortem examinations in individuals with confirmed SARS-CoV-2 infection who had died at the Ludwig-Maximilian University Hospital in Munich. Until autopsy, bodies were stored at + 4 °C for up to 78 h. The eyelids of the deceased were closed immediately after death in order to reduce postmortal evaporation of conjunctival fluid. Conjunctival swabs were taken from the right eye before autopsy. To obtain an adequate amount of conjunctival fluid, the deep fornix was wiped out. Thereafter, approximately 2 ml of vitreous was collected by intravitreal biopsy (IVB) directly through the anterior part of the bulbar conjunctiva from the right eye under sterile conditions with a 27-gauge needle (B. Braun Melsungen AG, Melsungen, Germany). The posterior chamber was then restored with balanced salt solution (B. Braun Melsungen AG, Melsungen, Germany). During autopsy, a swab was obtained from the bronchial system. For all specimen collection, nylon flocked swabs with liquid Amies transport medium were used (eSwabs, Copan diagnostics, Brescia, Italy). Conjunctival swabs, IVBs, and bronchial swabs were sent immediately at room temperature to the Department of Virology and tested immediately for SARS-CoV-2 RNA by qRT-PCR. Only subjects with evidence of SARS-CoV-2 RNA in the bronchial swab were included in the study. Clinical reports were reviewed for pre-existing medical conditions. None of the deceased patients had a history of ocular disease or ocular surgery. Cause of death was determined at autopsy, which was performed according to published best practice [26]. This study was approved by the local Institutional Review Board (No. 20–245), and written informed consent was obtained from the next of kin.

### Detection and quantification of SARS-CoV-2 RNA

Fresh paired post-mortem conjunctival, bronchial, and vitreous samples from 20 different donors were analyzed using the fully automated Roche cobas® 6800/8800 system (Roche Diagnostics, Penzberg, Germany). For quantification, standard curves were generated in multiple replicates using a commercially available standard for calibration (Instand e.V., Düsseldorf, Germany). Viral loads of samples were calculated as SARS-CoV-2 ORF1ab copy numbers per 1 ml of swab medium or vitreous fluid, respectively. Positive and negative control samples were included in each run. These measurements were performed at the fully accredited diagnostic laboratory of the Department of Virology at the Pettenkofer Institute.

### Genome sequencing

Total RNA was extracted from bronchial samples using the DSP virus/pathogen Mini Kit on a Qiasymphony robot (Qiagen, Hilden, Germany). Amplicon pools spanning the whole virus genome were prepared using the ARTIC protocol multiplex PCR, subsequently converted to barcoded sequencing libraries with the Nextera XT kit (Illumina, San Diego, USA) and sequenced on an Illumina Hiseq1500 sequencer (Illumina, San Diego, USA) as described previously [27]. The sequenced amplicons were demultiplexed and consensus sequences were generated using the iVar pipeline. The pileup files served as input for the consensus sequence generation within iVar where only variants that had a minimum read depth of 20 and a minimum frequency of 0.9 were considered. Phylogenetic analyses were achieved with the web and analysis platform Auspice using the SARS-Cov-2 build (https://github.com/nextstrain/ncov) and the bioinformatic toolkit augur. The consensus sequences and meta data for the samples were uploaded to the GISAID repository. GISAID accession numbers for the cases of this study are shown in Table 2.

### Statistical analysis

Statistical analyses were performed using Microsoft Excel version Microsoft Office 365 (Microsoft, Redmond, USA) and GraphPad Prism version 9 software (GraphPad Software, San Diego, USA). Bivariate correlation analyses between viral load in bronchial samples and viral load in conjunctiva and vitreous respectively were performed using the Spearman rank correlation test. The Spearman rank correlation is a non-parametric test and the Spearman rank correlation coefficient, i.e., Spearman’s Rho (r) measures the strength and direction of association between two ranked variables. *P* values ≤ 0.05 were considered statistically significant.

## Results

IVBs and conjunctival swabs were obtained during autopsy from 20 individuals with confirmed SARS-CoV-2 RNA in bronchial swabs. Patient characteristics are summarized in Table 1.

**Table 1:** Demographics and clinical characteristics

Pat. ID	Sex	Age	Positive for (in days)	ICU	Time until autopsy (in hours)	Cause of death
1	F	53	18	y	39	Intracranial bleeding
2	M	95	2	n	27	Sepsis
3	M	66	2	y	52	Cardiorespiratory failure
4	M	72	8	n	46	Respiratory failure
5	F	86	6	n	42	Pulmonary embolism
6	M	95	6	n	69	Respiratory failure
7	F	83	10	n	19	Respiratory failure
8	F	80	12	n	68	Respiratory failure
9	F	77	5	n	28	Cardiorespiratory failure
10	M	84	4	n	41	Respiratory failure
11	M	81	26	y	38	Respiratory failure
12	M	72	9	y	70	Respiratory failure
13	M	73	34	y	68	Multiorgan failure
14	M	59	94	y	31	Multiorgan failure
15	F	81	2	n	33	Sepsis
16	M	59	19	y	26	Multiorgan failure
17	M	52	28	y	46	sepsis
18	M	62	19	y	36	Respiratory failure
19	M	60	12	n	78	Pulmonary embolism
20	M	45	11	y	23	Sepsis

The median age at death of the study participants was 63 (44–95) years. Six (30%) of the deceased patients were female. In terms of comorbidities, 16 (80%) had known coronary artery disease, 16 (80%) had hypertension, 10 (50%) had chronic renal failure, nine (45%) had dyslipidemia, five (25%) had type II diabetes mellitus, four (20%) had dementia, three (15%) had COPD, two (10%) had Parkinson’s disease, and two (10%) had a post-organ-transplant condition. Ten patients (50%) were treated in the intensive care unit (ICU) at the time of death. The median time of positivity for SARS-CoV-2 RNA in nasopharyngeal swabs was 10 days (2–94) and the median time from exitus to autopsy was 44 (19–78) h. Causes of death were respiratory failure in eight patients (40%), sepsis in four (20%), multiorgan failure in three (15%), cardiorespiratory failure in two (10%), pulmonary embolism in two (10%), and intracranial hemorrhage in one (5%) of the patients. Neither specific ocular symptoms related to COVID-19 nor previously known ocular comorbidities or surgical procedures were listed in all the medical records.

**Fig. 1:**
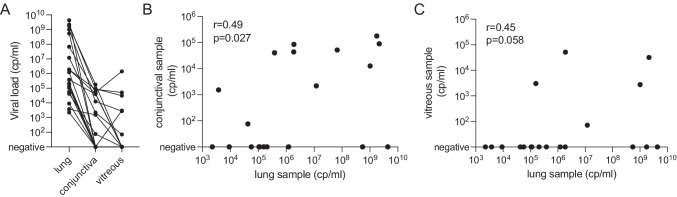
Viral loads of SARS-CoV-2 in conjunctival swabs and vitreous samples. (**a**) Viral loads are shown as SARS-CoV-2 ORF1ab copy numbers per ml for bronchial, conjunctival and vitreous samples. Results from the same patient are connected by a line. (**b** and **c**) Viral load levels of conjunctival and vitreous samples are shown in relation to bronchial samples. Spearman rank correlation tests are indicated

SARS-CoV-2 RNA was detected in 10 (50%) conjunctival swabs and 6 (30%) IVBs (Fig. 1a). A higher proportion of patients with a positive conjunctival test for SARS-CoV-2 RNA also had a positive IVB result. Thus, among the 10 patients with positive conjunctival swab, SARS-CoV-2 RNA was detected in the vitreous of 5 patients, but only one patient with a negative conjunctival swab had a positive IVB.

Viral load in bronchial samples was positively correlated with viral load in conjunctiva (Spearman rank correlation test; *r* = 0.49, *p* = 0.027; Fig. 1b) but not the vitreous (r = 0.45, p = 0.058, Fig. 1c). Patients in the ICU were positive for SARS-CoV-2-RNA for more days on average than patients in the normal ward (Mean 26 days vs. 6.7 days) and a larger fraction of ICU patients tested positive for SARS-CoV-2-RNA in conjunctival swabs or IVBs (80% vs 30%).

**Fig. 2:**
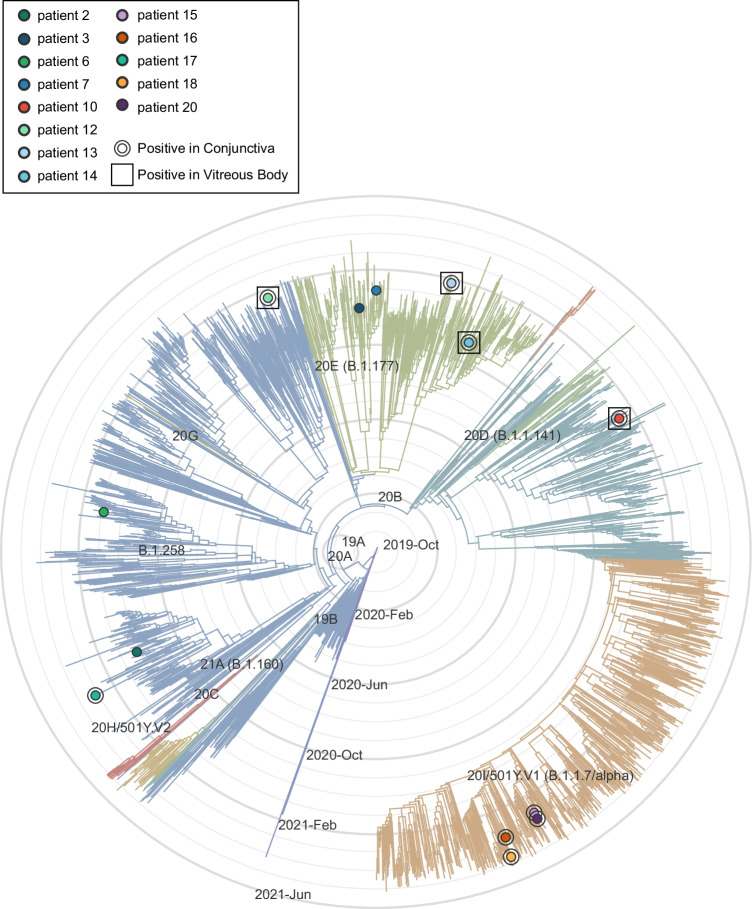
Phylogenetic relationship of SARS-CoV-2 post-mortem samples and samples from the Munich Metropolitean Area. Time-resolved maximum likelihood phylogeny of 2625 SARS-CoV-2 genomes from the larger Munich area obtained between March 2020 and February 2021. Nextstrain/Pangolin/WHO nomenclature clades are indicated above the main branches. The sequences of the cases in this study are indicated as colored dots. Cases with presence of SARS-CoV-2 RNA in the conjunctiva are marked by a black circle, and cases with detection of SARS-CoV-RNA in the vitreous body are indicated by a square. GISAID accession numbers for the cases of this study are shown in Table 2

To analyze if the presence of SARS-CoV-2 RNA in the eye is associated with infection by specific SARS-CoV-2 variants such as the recently emerged variant of concern (VOC), B.1.1.7/alpha, we performed SARS-CoV-2 whole genome sequencing from respiratory samples of the deceased patients. We obtained near full-length sequences (> 95% genome coverage) for 13 of these cases that were subsequently used for phylogenetic analysis. The distribution of cases largely reflected the frequency of circulating lineages in the Munich area at that time (Fig. 2). None of the four cases who were infected with VOC alpha had a SARS-CoV-2-RNA-positive IVB result, whereas four of the nine cases infected with a non-VOC strain tested positive for viral RNA in the IVB. Hence, there was no association between the presence of SARS-CoV-2 RNA in IVBs and infection with the VOC alpha. Results of SARS-CoV-2 RNA detection, quantification, and whole genome sequencing are summarized in Table 2.

**Table 2:** Quantification of SARS-CoV-2 RNA and genome sequencing

Pat. ID	CT numeric bronchial samples	Viral load bronchial samples (cp/ml)	Conjunctiva	CT numeric conjunctiva	Viral load conjunctiva (cp/ml)	Vitreous body	CT numeric vitreous body	Viral load vitreous body (cp/ml)	Pangolin lineage	GISAID accession ID
1	30.39	156,813	-	NA	NA	+	36.22	3015	NA	NA
2	13.63	4,361,119,298	-	NA	NA	-	NA	NA	B.1.160	EPI_ISL_1751567
3	29.99	205,648	-	NA	NA	-	NA	NA	B.1.177	EPI_ISL_1751564
4	30.97	105,842	-	NA	NA	-	NA	NA	NA	NA
5	30.89	111,739	-	NA	NA	-	NA	NA	NA	NA
6	18.35	548,819,077	-	NA	NA	-	NA	NA	B.1.258	EPI_ISL_2095237
7	27.36	1,222,593	-	NA	NA	-	NA	NA	B.1.177	EPI_ISL_1752145
8	34.59	9101	-	NA	NA	-	NA	NA	NA	NA
9	36.65	2253	-	NA	NA	-	NA	NA	NA	NA
10	16.33	2,157,884,964	+	31.21	89,952	+	32.75	31,674	B.1.1.141	EPI_ISL_1752180
11	31.89	56,735	-	NA	NA	-	NA	NA	NA	NA
12	21.44	67,587,939	+	32.03	51,599	+	27.14	1,419,189	B.1.177	EPI_ISL_1752212
13	26.72	1,886,556	+	31.29	85,205	+	32.04	51,250	B.1.177	EPI_ISL_1751846
14	24.00	11,921,143	+	36.69	2193	+	41.59	71	B.1.177	EPI_ISL_1751511
15	16.64	1,748,952,174	+	30.20	178,365	-	NA	NA	B.1.1.7	EPI_ISL_2094844
16	28.50	379,585	+	33.50	40,391	-	NA	NA	B.1.1.7	EPI_ISL_2095010
17	32.32	42,391	+	41.50	76	-	NA	NA	B.1.160	EPI_ISL_1752358
18	35.89	3771	+	37.20	1537	-	NA	NA	B.1.1.7	EPI_ISL_2095002
19	17.44	1,016,934,884	+	34.11	12,600	+	36.36	2761	NA	NA
20	26.75	1,848,583	+	32.78	43,556	-	NA	NA	B.1.1.7	EPI_ISL_2094634

*CT* cycle threshold, *NA* not applicable

## Discussion

This autopsy study investigated whether SARS-CoV-2 RNA could be detected in post-mortem samples of conjunctiva and vitreous of patients who had died from COVID-19. Since COVID-19 was first described in December 2019, ocular involvement in COVID-19 patients has been reported several times, and the conjunctiva and lacrimal fluid have been considered as a possible viral reservoir [3, 28–30]. The most common ophthalmological symptoms in COVID-19 are dry, watery, and painful eyes and photophobia [31]. Some studies have also described ischemic retinal changes in patients after infection with SARS-CoV-2 [32–35]. However, to date, it is still unclear how SARS-CoV-2 can target the eye and how frequently ocular involvement occurs in patients with COVID-19.

In the present study, we detected SARS-CoV-2 RNA in 50% of the examined conjunctival specimens and in 30% of the examined IVBs. Our results are in accordance with previous studies that found SARS-CoV-2 RNA within the eye [18, 19, 24]. In other studies, SARS-CoV-2 RNA was not detected in conjunctiva and vitreous [17, 20, 21, 23, 36]. One possible reason for the negative results in these studies may have been the small sample size. In our comparatively large post-mortem cohort of 20 deceased individuals, we found SARS-CoV-2 RNA in 10 conjunctival swabs and in 6 vitreous samples. There was a significant positive correlation between viral load in bronchial samples and viral load in conjunctiva, suggesting that conjunctival involvement is more likely in cases of severe COVID-19. A similar trend was observed for the association between viral load in bronchial samples and viral load in vitreous samples, which was not statistically significant, however, likely due to the overall low number of positive vitreous samples.

Conjunctival swab and IVBs were performed before autopsy and in a sterile manner. While the conjunctival swab was taken from the protected fornix, the puncture for IVB was performed directly through the anterior part of the bulbar conjunctiva. Due to post-mortem evaporation, virtually no tear fluid was present at this site at the time of puncture. These measures were intended to reduce the possibility of contamination of the vitreous samples with viral RNA by entrained tear fluid. While contamination of the vitreous by the puncture procedure cannot be ruled out completely, our results suggest that the risk is probably low. For example, in one case, the vitreous sample was positive, while the corresponding conjunctival sample was negative. In another case, a higher viral load was detected in the vitreous than in the corresponding conjunctival sample. In five subjects, the vitreous samples were negative, although viral RNA, and in some cases even high viral loads, were detected in the conjunctival samples. These results argue against accidental carryover of viral material from the conjunctiva into the interior of the eye during vitreous sample collection.

We did not find any evidence of COVID-19-related central nervous system (CNS) inflammatory disease in our cohort, which has been suggested as a possible starting point of ocular infection [23]. SARS-CoV-2 invades its host cells by binding to ACE-2 on the cell surface [37], which has been detected in ocular tissues, e.g., in conjunctiva and cornea, but also in the retina [13, 15, 16]. Given the expression of ACE-2 in the eye, it seems possible that SARS-CoV-2 may infect the conjunctiva directly from the outside and enter the interior of the eye via ACE-2. Our results support this hypothesis. It should be noted, however, that we analyzed only viral RNA and that detection of viral RNA does not necessarily imply the presence of potentially infectious viral particles. In this study, we chose PCR as a method with high sensitivity to detect even small amounts of viral RNA in the ocular region. Further studies using alternative detection methods (e.g., immunohistochemistry, electron microscopy) are needed to corroborate our results and investigate whether viral proteins and (infectious) SARS-CoV-2 particles are present in the eye during COVID-19 disease.

In our study, SARS-CoV-2 RNA could be detected intraocularly up to 78 h after death and up to 94 days after the first positive test. A recent report by Koo et al. showed that SARS-CoV-2 RNA was present in the aqueous humor of asymptomatically infected subjects despite negative nasal swab tests [24]. This raises the question whether and how long SARS-CoV-2 can persist in immune-privileged niches like the eye without causing any symptoms. Similarly, little information is available about the development and persistence of ocular symptoms in patients with COVID-19 and particularly post-COVID-19 syndrome. These questions need to be addressed in further prospective studies.

The possibility of altered tissue tropism of new and future SARS-CoV-2 variants, which may lead to different organ manifestations, must be considered [38]. To address this question, we performed viral sequencing and phylogenetic analyses to determine the viral lineages of our cases. The distribution corresponded to the frequency of circulating SARS-CoV-2 variants in the Munich area at that particular time. In particular, the proportion of cases in which SARS-CoV-2 RNA was detected in the vitreous was not increased in patients infected with the “variant of concern” (VOC)-alpha virus, suggesting that this variant does not have increased tropism for intraocular tissue.

Although the infectivity of ocular fluids during COVID-19 disease still needs to be verified in cell culture-based studies, our results suggest that ophthalmologists should wear appropriate protective equipment when performing ocular procedures on COVID-19 patients, such as pars plana vitrectomy or corneal transplantation. In addition, it seems important to pay special attention to personal protection and strictly adhere to appropriate hygiene measures not only during surgical procedures but also in the outpatient setting to prevent infections and the spread of SARS-CoV-2. Therefore, preventive management concepts analogous to the management of patients with keratoconjunctivitis epidemica should be considered.

In conclusion, we found that genetic material of SARS-CoV-2 is present in the conjunctiva and vitreous of individuals severely affected by COVID-19. Further studies are needed to better understand the pathology and significance of ocular involvement in COVID-19.

## Data Availability

All datasets on which the conclusions of the paper rely are available and stated in this manuscript.
